# Incidence of Antibiotic Treatment Failure in Patients with Nursing Home-Acquired Pneumonia and Community Acquired Pneumonia

**DOI:** 10.3390/idr13010006

**Published:** 2021-01-05

**Authors:** Mariana Lopes, Gonçalo Alves Silva, Rui Filipe Nogueira, Daniela Marado, João Gonçalves, Carlos Athayde, Dilva Silva, Ana Figueiredo, Jorge Fortuna, Armando Carvalho

**Affiliations:** 1 Infectious Diseases Department, Coimbra Hospital and Universitary Centre, 3004-561 Coimbra, Portugal; 2 Nephrology Department, Coimbra Hospital and Universitary Centre, 3004-561 Coimbra, Portugal; 11591@chuc.min-saude.pt; 3 Internal Medicine Department, Coimbra Hospital and Universitary Centre, 3004-561 Coimbra, Portugal; 13150@chuc.min-saude.pt (D.M.); 26860@chuc.min-saude.pt (J.G.); cathayde@chuc.min-saude.pt (C.A.); 21750@chuc.min-saude.pt (D.S.); anafigueiredo@chuc.min-saude.pt (A.F.); jorge.fortuna@chuc.min-saude.pt (J.F.); acarvalho@chuc.min-saude.pt (A.C.)

**Keywords:** nursing home-acquired pneumonia, community-acquired pneumonia, antimicrobial drug resistance, epidemiology, treatment failure

## Abstract

Purpose: Nursing home-acquired pneumonia (NHAP) patients are at higher risk of multi-drug resistant infection (MDR) than those with community-acquired pneumonia (CAP). Recent evidence suggests a single risk factor for MDR does not accurately predict the need for broad-spectrum antibiotics. The goal of this study was to compare the rate antibiotic failure between NHAP and CAP patients. Methods: Demographic characteristics, co-morbidities, clinical and laboratory variables, antibiotic therapy, and mortality data were collected retrospectively for all patients with pneumonia admitted to an Internal Medicine Service between April 2017 and April 2018. Results: In total, 313 of 556 patients had CAP and 243 had NHAP. NHAP patients were older, and were more likely to be dependent, to have recent antibiotic use, and to experience treatment failure (odds ratio (OR) 1.583; 95% CI 1.102–2.276; *p* = 0.013). In multivariate analysis, patient’s origin did not predict treatment failure (OR 1.083; 95% CI 0.726–1.616; *p* = 0.696). Discussion: Higher rates of antibiotic failure and mortality in NHAP patients were explained by the presence of other risk factors such as comorbidities, more severe presentation, and age. Admission from a nursing home is not a sufficient condition to start broader-spectrum antibiotics.

## 1. Introduction

Pneumonia is one of the major causes of death in Portugal, resulting in 57.7 deaths for every 100,000 habitants [1]. Worldwide, the incidence of pneumonia in residents of long-term care facilities is estimated to be 10 times higher than in the general population, whereas the rate of hospital admissions is 30 times higher [2], with pneumonia being the most frequent infectious cause of hospital admission in nursing home patients [3]. Mortality is also higher in patients with nursing home-acquired pneumonia (NHAP) at about 20%, which is a similar rate to hospital-acquired pneumonia (HAP) [4].

NHAP was incorporated into the concept of healthcare-associated pneumonia (HCAP) when it was introduced to the American Thoracic Society and the Infectious Diseases Society of America (ATS/IDSA) 2005 clinical practice guidelines for hospital-acquired, ventilator-associated, and healthcare-associated pneumonia in adults. The HCAP classification was based on two large U.S. studies [5] that showed an elevated risk of infection with multidrug-resistant organisms (MDRO) in patients with pneumonia and recent contact with the healthcare system.

In the proposed classification, a patient was considered to have healthcare-associated pneumonia if they had at least one of the following risk factors [6]: hospitalization for more than 2 days in an acute care hospital in the previous 90 days; NHAP; intravenous antibiotic therapy, chemotherapy, or wound care in the previous 30 days; attending a hospital or hemodialysis clinic; and immunosuppression.

However, the concept of HCAP faced criticism as subsequent retrospective studies in other countries showed differing rates of MDR infections, with Spain and the UK reporting much lower incidences of MDR bacteria in HCAP patients than in the USA [5,7]. Additionally, prospective studies designed to evaluate the HCAP concept showed that the presence of a single risk factor, such as residence in a nursing home, did not accurately predict the need for broad-spectrum antibiotics [8], and scores that took into account the number of MDR risk factors outperformed the HCAP definition [9,10,11,12,13,14]. Because of this, HCAP was removed from the most recent ATS/IDSA community-acquired pneumonia guidelines in 2019 [15] and was likewise not included in the current recommendations by the Joint Taskforce of the European Respiratory Society and European Society for Clinical Microbiology and Infectious Diseases [16].

However, it is clear that the incidence of MDR infection in non-hospitalized patients varies between countries [5], and the changing microbiome of the nursing home and healthcare facilities justifies continued surveillance for the risk of resistant infections and the need to increase the spectrum of empirical coverage in these patients. Whereas studies elsewhere in Europe showed a low prevalence of MDR organisms in nursing home patients, a recent retrospective study of nursing home-acquired pneumonia patients in Portugal, by Pereira et al. [17], showed a high rate of potentially MDR organisms, with a pattern that more closely resembled the original HCAP studies in the USA.

The goal of this study was to compare the clinical presentation, outcomes, microbiological identification, and patterns of antibiotic resistance between patients with community-acquired pneumonia and those with nursing home-acquired pneumonia in order to determine if there currently exists a significant enough difference to justify a broader-spectrum of empirical coverage in these patients.

## 2. Methods

### 2.1. Study Design

In this retrospective observational study, all patients admitted to the Internal Medicine Service with a diagnosis of pneumonia from 01 April 2017 to 30 April 2018 were selected for inclusion. Patients with hospitalization for more than 48 h in the preceding 10 days were considered to have hospital-acquired pneumonia and were excluded. The patient population was divided into 2 groups: NHAP and CAP. Baseline demographic characteristics, comorbidities, functional status, clinical and laboratory markers of pneumonia severity at presentation, chosen course of empiric antibiotics and the need to change antibiotic therapy, and mortality and readmission outcomes were collected. The study protocol was reviewed and approved by the Ethics Committee of Coimbra Hospital and Universitary Centre (CHUC). Informed consent was waived due to the retrospective study design and complete anonymization of patient data.

### 2.2. Definitions

The patients were defined as having pneumonia if they presented with a clinical history compatible with the diagnosis of pneumonia (at least one of the following: temperature ≥37.8 °C, new-onset cough, leukocytosis, or leukopenia) plus confirmation of a new pulmonary consolidation on chest radiography.

The NHAP group included residents of nursing homes, residents of medium-term and rehabilitation units, and residents of long-term and maintenance units. Prior antibiotic use was defined as a prescribed antibiotic regimen in the previous 6 months.

Pneumonia severity was determined using the CURB-65 score (new-onset confusion, blood urea nitrogen > 19 mg/dL, respiratory rate ≥30 breaths per minute, blood pressure < 90 mm Hg systolic, or diastolic blood pressure < 60 mmHg, and age ≥65 years).

Functional status was assessed through the Katz scale and patients were classified into 3 groups: independent (6 points), partially dependent (1–5 points), and dependent (0 points).

Empirical antibiotic regimens were defined as covering for MDR pathogens if they included an antibiotic drug known to cover for methicillin-resistant *Staphylococcus aureus* (MRSA), *Pseudomonas aeruginosa*, and *Enterococcus* species. The antibiotics included in this group were vancomycin, ceftazidime, trimethoprim/sulfamethoxazole, fluoroquinolones, piperacillin-tazobactam, and meropenem.

Antibiotic failure was defined as death during the course of antibiotic therapy or a change in antibiotic regimen due to a lack of clinical improvement.

### 2.3. Clinical and Laboratory Variables

Venous blood samples obtained at admission were analyzed. Leukocyte count, neutrophil count, creatinine, urea, hematocrit, sodium, albumin, lactate dehydrogenase (LDH), glucose, N-terminal prohormone of brain natriuretic peptide (NT-proBNP), and C-reactive protein values were collected. Arterial blood gas was used to determine PaO2/FiO2, PaCO2, pH, and lactate values.

### 2.4. Microbiological Evaluation

Data from blood and sputum cultures were collected, as well as urinary antigen testing for *Streptococcus pneumoniae* and *Legionella* species.

### 2.5. Clinical Outcomes

The primary outcome of interest was antibiotic failure. Readmission in the first 30 days, mortality during hospitalization, and mortality at day 30 after discharge were secondary outcomes.

### 2.6. Statistical Analysis

Shapiro–Wilk’s *W*-test was calculated to test for normal distribution of continuous variables. Parametric variables are presented as mean (± standard deviation), and non-parametric variables are presented as median and interquartile range (IQR). For inter-group comparisons where the data were normally distributed, continuous variable comparisons were made using Student’s *t*-test, otherwise the non-parametric Mann–Whitney *U* test was used.

Categorical variables were compared using the chi-squared test or Fisher’s exact test if more than 20% of the expected cell frequencies were <5.

Univariable logistic regression analysis was used to assess potential risk factors for antibiotic failure and multidrug resistance. Variables found to be predictive of antibiotic failure and variables found to be predictive of multidrug resistance in univariable analysis were inserted into a multivariable logistic regression model. The association of each risk factor with the outcome of interest was denoted by the odds ratio (OR) and corresponding 95% confidence intervals (CI).

Statistical significance was assumed for *p* < 0.05. Statistical analysis was performed using SPSS Statistics version 25.0 (IBM Corp. Armonk, NY, USA).

## 3. Results

### 3.1. Patient Characteristics

A total of 782 patients with a diagnosis of pneumonia admitted to the Internal Medicine Service were analyzed, of which 556 fulfilled selection criteria. CAP was found in 313 (56.3%), and the remaining 243 (43.6%) patients had NHAP. The baseline characteristics of the patients with CAP and NHAP are presented in Table 1.

There was no difference in sex distribution between the two groups. Compared with the CAP group, NHAP patients had a higher median age (84 vs. 87, *p* < 0.001), and they were also more likely to be dependent for activities of daily living on the basis of the Katz scale (31% vs. 67.1%, *p* < 0.001). There was no difference in prevalence of comorbidities between the two groups, with the exception of cerebrovascular disease (CVD), which was more common in NHAP (14.4% vs. 30.5%, *p* < 0.001), and chronic liver disease, which was more common in CAP (2.6% vs. 0.0%, *p* < 0.011). Patients admitted from nursing homes were more likely to have taken an antibiotic in the previous 6 months (40.3% vs. 58%, *p* < 0.001).

### 3.2. Initial Clinical Features and Severity at Presentation

Clinical features and severity stratification at admission for CAP and NHAP patients are shown in Table 1. NHAP patients were more likely to present with a more severe CURB-65 score (*p* < 0.001), with 66.3% of CAP patients compared to 82.3% of NHAP patients presenting with a score ≥3. This increased severity was also noted in a higher incidence of respiratory failure in the NHAP group (73.6% vs. 83.1%, *p* = 0.008). Median temperature and systolic blood pressures were higher in the CAP group. Although there was a trend towards more frequent vasoactive amine and non-invasive ventilation use in CAP compared to NHAP patients, this was not statistically significant.

The distribution of laboratory variables at admission is also presented in Table 1. Urea values were higher in the NHAP group (61 vs. 67.2 mg/dL, *p* = 0.029). Albumin value was lower in this group (34 vs. 32 g/L, *p* < 0.001). Other variables, including arterial blood gas analysis, NT-proBNP, and C-reactive protein showed no difference between groups.

### 3.3. Isolation of Microorganisms

Blood culture was the most common test used in an attempt to establish an etiological diagnosis in both CAP and NHAP (58.8% vs. 61.3%, *p* = 0.546) (Table 1). Sputum cultures were collected more frequently from NHAP patients (10.5% vs. 16.9%, *p* = 0.029). Pneumococcal and *Legionella* urinary antigen tests were more often requested in CAP patients (13.1% vs. 6.6%, *p* = 0.012 for the pneumococcal antigen, and 12.8% vs. 6.2%, *p* = 0.01 for the *Legionella* antigen).

At least 1 microorganism was isolated in culture samples of 66 patients (11.9%), of which 32 had CAP and 34 had NHAP (Table 2). In the majority of cases, microorganisms were identified through blood cultures, but sputum culture showed a higher yield compared to blood culture when taking into account the total number of samples (34/74 vs. 40/333).

The most frequently identified microorganism was *Staphylococcus aureus*, followed by *Escherichia coli*, in either group. *Haemophilus influenzae* was detected only in CAP patients. *Pseudomonas* species were more frequent in the NHAP group (Table 2).

### 3.4. Empirical Antibiotic Therapy

Patients in the NHAP group were more likely to receive empirical antibiotic regimens with MDRO coverage than those in the CAP group (28.8% 90 vs. 43.0% 104, *p* < 0.001) (Table 3). The preferred non-MDRO regimens were a combination of amoxicillin-clavulanate plus azithromycin (45.8% vs. 21.8%) or ceftriaxone plus azithromycin (13.1% vs. 17.7%). Levofloxacin (16.9% vs. 18.9%) and piperacillin-tazobactam (7.7% vs. 17.7%) were the most frequent MDRO regimens used.

Antibiotic susceptibility testing showed that 47% of isolated microorganisms were multidrug-sensitive. Microorganisms isolated from NHAP patients were more likely to be multidrug- or extensively drug-resistant (65.7% vs. 34.3%, OR 3.485, *p* = 0.014) (Table 2).

### 3.5. Clinical Outcomes

The clinical outcomes of patients with NHAP and CAP are shown in Table 4. The NHAP group, compared to CAP, had a higher frequency of antibiotic failure (36.4% vs. 26.5%, *p* = 0.013), readmission in 30 days, and death until 30 days after discharge. The origin of the patient had a statistically significant relationship with therapeutic failure, with an OR of 1.583 (95% CI 1.102–2.276; *p* = 0.013).

Baseline characteristics of the patients that were found to predict therapeutic failure in univariate logistic regression were inserted into a multivariable logistic regression model, presented in Table 5. When controlled for age, functional status, and previous antibiotic use, NHAP no longer predicted antibiotic failure (OR 1.083; 95% CI 0.726–1.616; *p* = 0.696).

In a multivariable logistic regression model of culture-positive patients, NHAP also did not predict identification of MDR microorganisms when controlled for patient characteristics that predicted MDRO in univariate analysis (OR 1.947; 95% CI 0.653–5.802; *p* = 0.232), as seen in Table 5.

## 4. Discussion

The NHAP patients in this study were older, had worse functional status (61.7% dependent patients), and had a higher prevalence of CVD than CAP patients. They presented clinically with more severe pneumonia, with a higher CURB-65 score and a higher rate of respiratory failure. A higher prevalence of CVD in NHAP patients has also been recorded by Kang et al. [18], who identified a correlation with risk of aspiration, justifying the worse clinical presentation. Albumin levels at admission were also lower in these patients, and blood urea levels were higher, differences that have been associated in the literature with poor functional status as these patients are more likely to have baseline poor nutrition and dehydration according to Nakagawa et al. and Martínez-Moragón et al. [19,20].

The proportion of patients with the causative pathogens identified was low (11.9% of patients) in comparison with previous studies that report a positive microbiological diagnosis in between 24 and 43% of patients [11,21,22,23,24]. This discrepancy can be explained at least partly by the relatively low use of sputum cultures and urinary antigen testing. *Enterobacteriaceae* species (especially *Escherichia coli*), followed by *Staphylococcus aureus*, were the most common bacterial isolates in this study, with a low identification of *Streptococcus pneumoniae* and *Haemophilus influenzae*. This differs from expected values in the literature, as a meta-analysis by Chalmers et al. describes a predominance of *Streptococcus pneumoniae* in the CAP group and a predominance of *Enterobacteriaceae* in the HCAP group [25]. However, our results are similar to another Portuguese study of NHAP patients that reported a predominance of *Staphylococcus aureus* with a very low rate of *Streptococcus pneumoniae* as well as a significant percentage of *Escherichia coli* isolates [17], and in a systematic review by Dhawan et al., the most common bacteria isolated in NHAP patients were *Staphylococcus aureus* and enteric Gram-negative bacilli [26]. In the CAP group, there was also a low incidence of *Streptococcus pneumoniae* with a reciprocal higher than expected relative prevalence of *Escherichia coli* and *Pseudomonas aeruginosa.* While the promotion of vaccination against invasive pneumococcal disease in older people might explain in part the lower-than-expected identification of this pathogen in this population, the relatively low use of sputum cultures and urinary antigen testing are also likely to have affected the identification of *Streptococcus pneumoniae* and *Haemophilus influenzae* infections. Since the decision to draw cultures was based on the individual clinician’s assessment, it is also likely that the choice to not pursue etiological diagnosis in less severe patients might contribute to an underdetection of *Streptococcus pneumoniae* and *Haemophilus influenzae*.

In our study, just over half of microorganisms isolated were multidrug- or extensively drug-resistant, and the patients in the NHAP group were at higher risk for MDRO than those in the CAP group (OR 2.86, *p* = 0.032). In-hospital mortality was also 1.6 times higher in the NHAP group (*p* = 0.013). The association between NHAP and an increased risk for MDRO has consistently been reported in the literature [19,25,27,28,29,30], as well as an association with increased mortality [18,24,31,32]. Although a review by Falcone et al. claimed a relationship between the microbiology patterns in HCAP patients and higher mortality rate [33], other studies directed at predicting MDRO in HCAP patients such as the study by Gross et al., which found that isolation of MDRO did not influence mortality when adjusted for age, CURB-65 score, and comorbidities [34].

The global mortality rate for NHAP patients in this study (32.5%) is higher when compared with other studies that showed mortality rates varying between 14.1 and 22.4% [19,21,27,29,35,36], which may be explained by broader criteria for detecting mortality (from admission to 30 days after discharge). In-hospital mortality was similar to the rates reported in the literature at 21.4% for NHAP patients and 13.4% for CAP patients.

The ability of NHAP, as well as other HCAP criteria, to predict MDRO infection and hence justify the use of broader spectrum antibiotics had been questioned since the adoption of the original HCAP recommendations, with reviews showing that the rate of MDRO infection in these patients varied significantly between countries [5]. A recent retrospective study of NHAP patients in Portugal by Pereira et al. [17] showed a high rate of potentially MDR organisms and recommended the use of broader antibiotic coverage. In our study, although there was a similar pattern of bacterial isolates and higher incidence of MDRO in the NHAP group, this difference disappeared when controlling for age and previous antibiotic use. The main outcome of interest in this study was the rate of antibiotic failure, which was also significantly higher in the NHAP group. However, in a logistic regression model controlling for factors also correlated with antibiotic failure, such as functional status, age, and previous antibiotic use, the NHAP group did not accurately predict antibiotic failure. These results are in accordance with the recent literature [18,26,27,34,37].

A multicenter prospective study concluded that not giving empirical antibiotic therapy advised to healthcare-associated pneumonia was independently associated with higher mortality [38]. Yet, a review elaborated by Ewig et al. concluded there was insufficient evidence for a relationship between adverse outcomes in NHAP patients and MDRO [4] and choosing an empirical regimen to cover MDRO in institutionalized patients did not show better outcomes either [4,7,21,25,27,34].

A few limitations need to be addressed. First, the data were collected retrospectively from a single institution. Because of this, the choice to take cultures and urinary antigens was left to the individual clinician, introducing a risk for bias towards identification of microorganisms only in severe patients. Particularly in CAP patients, the results may not be representative of the actual microbiological pattern. As blood cultures, which have an inherently low sensitivity in respiratory infections, were the most used method for isolation of microorganisms, it might have further contributed to a low percentage of identified microorganisms. To compensate for an expected low diagnostic yield, we defined the main study outcome as the presence of antibiotic failure, a combination of mortality during hospitalization, or a change in antibiotic treatment. This introduces a different possibility of bias, as more severe patients, such as those in the NHAP group, might be more likely to die independently of adequate antibiotic treatment. Lastly, prognostic scores such as the CURB-65 was used in this study to identify patients with more severe pneumonia. However, the utility of these scores is variable [15], with studies showing they lack discriminatory power in NHAP due to the higher baseline scores seen in these patients [24,29,39]. The Sequential Organ Failure Assessment (SOFA) score was recently shown to outweigh the CURB-65 and other prognostic scores in predicting mortality and admission to intensive care units [40]. Due to the retrospective nature of this study, data required to accurately calculate the SOFA score (such as the Glasgow coma scale) were not available for most patients.

## 5. Conclusions

The present study demonstrates that nursing home residents with pneumonia are at higher risk for antibiotic failure and antibiotic resistance, but this increased risk can be accounted for by the presence of comorbidities, age, and previous antibiotic use. This is in line with the current IDSA recommendations, reinforcing the need for selective use of broad-spectrum antibiotics in patients with pneumonia that takes into account a combination of risk factors for MDRO, as admission from a nursing home is not a sufficient condition to initiate empirical broad-spectrum antibiotics.

## Figures and Tables

**Table 1:** Baseline demographic, comorbidities, previous antibiotic use, initial clinical features, and laboratory variables compared between community-acquired pneumonia (CAP) and nursing home-acquired pneumonia (NHAP) groups.

	CAP	NHAP	
Male, *n* (%)	154 (49.2)	100 (41.2)	*p* = 0.059
Median age, years (IQR)	84 (77–88.5)	87 (83–91)	***p* < 0.001**
Katz scale, *n* (%)			***p* < 0.001**
Independent	99 (31.6%)	10 (4.1)	
Partially dependent	117 (37.4%)	70 (28,8)	
Dependent	97 (31%)	163 (67.1)	
Comorbidities, *n* (%)			
Heart failure	160 (51.1)	123 (50.6)	*p* = 0.907
Atrial fibrillation	100 (31.9)	69 (28.4)	*p* = 0.366
Chronic renal disease	67 (21.4)	59 (24.3)	*p* = 0.422
Diabetes mellitus	103 (32.9)	83 (34.2)	*p* = 0.757
COPD	44 (14.1)	27 (11.1)	*p* = 0.302
Hypertension	226 (72.2)	175 (72.0)	*p* = 0.961
CVD	45 (14.4)	74 (30.5)	***p* < 0.001**
Chronic liver disease	8 (2.6)	0 (0.0)	***p* = 0.011**
Active malignancy	35 (11.2)	15 (6.2)	***p* = 0.041**
Immune suppression	13 (4.2)	12 (4.9)	*p* = 0.658
Antibiotic use (previous 6 months), *n* (%)	126 (40.3)	141 (58.0)	***p* < 0.001**
CURB-65, *n* (%)			***p* < 0.001**
0	5 (1.6)	5 (1.6)	
1	24 (7.7)	24 (7.7)	
2	76 (24.4)	76 (24.4)	
3	129 (41.3)	129 (41.3)	
4	65 (20.8)	65 (20.8)	
5	13 (4.2)	13 (4.2)	
Clinical features at admission			
Respiratory failure, *n* (%)	229 (73.6)	202 (83.1)	***p* = 0.008**
Pleural effusion, *n* (%)	64 (20.4)	42 (17.1)	*p* = 0.346
Temperature, °C (IQR)	37.2 (36.5–38.0)	37.0 (36.3–37.6)	***p* = 0.004**
Heart rate, bpm (IQR)	85.5 (75.0–101.0)	89.0 (76.0–102.0)	*p* = 0.370
Systolic blood pressure, mmHg (IQR)	122.5 (107.3–141.0)	116.5 (100.0–133.0)	***p* < 0.001**
Non-invasive ventilation, *n* (%)	25 (8.0)	14 (5.8)	*p* = 0.308
Vasoactive amines, *n* (%)	9 (2.9)	4 (1.7)	*p* = 0.345
Laboratory values			
Leukocyte count, cells/µL(IQR)	12,800 (9495–16,700)	13,000 (9900–18,400)	*p* = 0.081
Neutrophil count, cells/µL (IQR)	10,300 (7115–13,810)	10,990 (7600–15,420)	***p* = 0.037**
Creatinine, mg/dL (IQR)	1.02 (0.76–1.61)	1.05 (0.74–1.59)	*p* = 0.571
Urea, mg/dL (IQR)	61.0 (41.0–93.6)	67.2 (46.0–109.0)	***p* = 0.029**
Haematocrit, % (IQR)	37.0 (32.3–40.9)	35.7 (31.7–40.3)	*p* = 0.180
Sodium, mEq/L (IQR)	137.6 (135.0–140.9)	138.3 (133.0–142.3)	*p* = 0.531
Albumin, g/L (IQR)	34 (30–37)	32 (28–35)	***p* < 0.001**
LDH, U/L (IQR)	361.0 (246.0–521–5)	374.5 (249.3–542.5)	*p* = 0.627
Glucose, mg/dL (IQR)	132 (109–171)	138 (110–180)	*p* = 0.235
PaO_2_/FiO_2_ (IQR)	266.7 (233.7–300.6)	257.1 (206.7–300.0)	*p* = 0.080
PaCO_2_ (IQR)	40.0 (35.0–48.0)	41.0 (35.8–47.0)	*p* = 0.572
pH (IQR)	7.46 (7.41–7.49)	7.44 (7.39–7.48)	*p* = 0.254
Lactate, mmol/L (IQR)	1.2 (0.8–1.8)	1.3 (0.9–2.2)	*p* = 0.083
NT-proBNP, pg/mL (IQR)	3065.2 (838.3–9832.5)	2790.9 (889.5–10,675.0)	*p* = 0.956
C-reactive protein, mg/dL (IQR)	12.5 (5.6–20.9)	12.3 (6.7–19.5)	*p* = 0.848
Requested cultures and urinary antigen tests			
Blood culture, *n* (%)	184 (58.8)	149 (61.3)	*p* = 0.546
Sputum culture, *n* (%)	33 (10.5)	41 (16.9)	***p* = 0.029**
*S. pneumoniae* urinary antigen test, *n* (%)	41 (13.1)	16 (6.6)	***p* = 0.012**
Positive	4 (10.8)	2 (13.3)	*p* = 1.000
Legionella urinary antigen test, *n* (%)	40 (12.8)	15 (6.2)	***p* = 0.010**
Positive	1 (2.9)	0 (0.0)	*p* = 1.000

**Table 2:** Source of microorganism isolation, identification, and resistance pattern in the CAP and NHAP groups.

	Total	CAP	NHAP
Positive blood culture, *n* (%)	34 (51.5)	17 (53.1)	17 (50.0)
Positive sputum culture, *n* (%)	26 (39.4)	12 (37.5)	14 (41.2)
Positive blood and sputum culture, *n* (%)	6 (9.1)	3 (9.4)	3 (8.8)
Total	66	32	34
Gram-positive, *n* (%)			
*Streptococcus pneumoniae*	3 (4.5)	2 (6.3)	1 (2.9)
*Staphylococcus aureus*	18 (27.3)	8 (25.0)	10 (29.4)
Other *Streptococcus* species	4 (6.1)	2 (6.3)	2 (5.9)
Other *Staphylococcus* species	1 (1.5)	0 (0)	1 (2.9)
*Enterococcus faecalis*	1 (1.5)	1 (3.1)	0
Gram-negative, *n* (%)			
*Pseudomonas species*	6 (9.1)	1 (3.1)	5 (14.7)
*Klebsiella species*	8 (12.1)	4 (12.5)	4 (11.8)
*Escherichia coli*	11 (16.7)	6 (18.8)	5 (14.7)
*Haemophilus influenzae*	4 (6.1)	4 (12.5)	0 (0)
*Acinetobacter baumannii*	1 (1.5)	1 (3.1)	0 (0)
Polymicrobial, *n* (%)	9 (13.6)	3 (9.4)	6 (17.6)
Resistance pattern			
Multidrug-sensitive, *n* (%)	31 (47.0)	20 (61.8)	11 (36.1)
Multidrug-resistant, *n* (%)	20 (30.3)	9 (29.4)	11 (30.6)
Extensively drug-resistant, *n* (%)	15 (22.7)	3 (8.8)	12 (33.3)

**Table 3:** Empirical antibiotic therapy used in the CAP and NHAP groups.

	Total *	CAP	NHAP
**Non-MDRO coverage, *n* (%)**	**360**	**222 (70.9)**	**138 (57.0)**
Monotherapy			
Amoxicillin-clavulanate	40	15 (4.8)	25 (10.3)
Ceftriaxone	28	14 (4.5)	14 (5.8)
Cefuroxime	3	3 (1.0)	0 (0.0)
Azithromycin	2	1 (0.3)	1 (0.4)
Doxycycline	1	1 (0.3)	0 (0.0)
Combination therapy			
Amoxicillin-clavulanate + azithromycin	196	143 (45.8)	53 (21.8)
Ceftriaxone + azithromycin	84	41 (13.1)	43 (17.7)
Cefuroxime + azithromycin	3	2 (0.6)	1 (0.4)
Other combinations	3	2 (0.6)	1 (0.4)
**MDRO coverage, *n* (%)**	**195**	**91 (29.1)**	**104 (43.0)**
Monotherapy			
Trimethoprim/sulfamethoxazole	6	2 (0.6)	4 (1.6)
Ceftazidime	5	1 (0.3)	4 (1.6)
Ciprofloxacin	1	1 (0.3)	0 (0.0)
Levofloxacin	99	53 (16.9)	46 (18.9)
Meropenem	6	4 (1.3)	2 (0.8)
Combination therapy			
Trimethoprim/sulfamethoxazole + ceftazidime	3	1 (0.3)	2 (0.8)
Trimethoprim/sulfamethoxazole + piperacillin-tazobactam	2	1 (0.3)	1 (0.4)
Piperacillin-tazobactam	67	24 (7.7)	43 (17.7)
Piperacillin-tazobactam + vancomycin	2	2 (0.6)	0 (0.0)
Meropenem + vancomycin	1	1 (0.3)	0 (0.0)
Other combinations	3	1 (0.3)	2 (0.8)

* patient did not receive antibiotic therapy.

**Table 4:** Clinical outcomes compared between CAP and NHAP groups.

	CAP	NHAP	
Antibiotic failure, *n* (%)	83 (26.5)	88 (36.4)	***p* = 0.013**
Readmission (30 days), *n* (%)	44 (14.7)	49 (21.7)	***p* = 0.037**
Death, *n* (%)			
Admission until 30 days after discharge	61 (19.5)	79 (32.5)	***p* < 0.001**
During hospitalization	42 (13.4)	52 (21.4)	***p* = 0.013**
Days until death, *n* (IQR)	11 (5–19)	9 (4–16)	*p* = 0.165
Days of hospitalization, *n* (IQR)	8 (5–15)	6 (3–12)	*p* = 0.600

**Table 5:** Univariable and multivariable logistic regression of variables found to predict antibiotic failure and found to predict isolation of multi- or extensively drug-resistant microorganisms.

Antibiotic Failure	Univariable Logistic Regression			Multivariable Logistic Regression		
	OR	95% CI	*p*-Value	OR	95% CI	*p*-Value
NHAP	1.583	1.102–2.276	0.013	1.083	0.726–1.616	0.696
Age	1.042	1.019–1.065	0.000	1.031	1.008–1.055	**0.008**
Antibiotic use (previous 6 months)	1.498	1.040–2.158	0.030	1.172	0.794–1.731	0.425
Katz scale	0.835	0.772–0.903	0.000	0.867	0.792–0.949	**0.002**
Isolation of MDRO	Univariable Logistic Regression			Multivariable Logistic Regression		
	OR	95% CI	*p*-Value	OR	95% CI	*p*-Value
NHAP	2.858	1.083–7.539	0.034	1.947	0.653–5.802	0.232
Age	1.055	1.006–1.107	0.028	1.065	1.011–1.121	**0.018**
Antibiotic use (previous 6 months)	4.143	1.429–12.012	0.009	4.275	1.316–13.890	**0.016**

## Data Availability

The data presented in this study are available on request from the corresponding author. The data are not publicly available due to ethical and privacy issues.
